# Supplementary material for: First stage progression in women with spontaneous onset of labor: A large population-based cohort study
Source: PLoS One. 2020 Sep 25;15(9):e0239724. doi: 10.1371/journal.pone.0239724 (PMC7518577; doi:10.1371/journal.pone.0239724)
Supplement: S1 Table — (DOCX) [file pone.0239724.s001.docx]

**S1 Table. Maternal, labor and fetal characteristics for Target population cohort**

|  | **Parity=0**  **n=56 392** | | | **Parity=1**  **n=30 703** | | | **Parity=2+**  **n=14 635** | | |
| --- | --- | --- | --- | --- | --- | --- | --- | --- | --- |
| **Characteristics** |  | % | N |  | % | N |  | % | N |
| **Age** years, mean (SD) | 29.5 (4.99) |  | 56 352 | 31.6 (4.65) |  | 30 687 | 34.1 (4.63) |  | 14 634 |
| **Maternal height cm** mean (SD) | 166.4 (6.48) |  | 55 829 | 166.4 (6.51) |  | 30 434 | 165.6 (6.54) |  | 14 497 |
| **BMI.** kg/m^2^ at first visit antenatal clinic | 23.6 (4.03) |  | 54 310 | 24 (4.37) |  | 29 545 | 25.3 (4.73) |  | 14 118 |
| **Family situation** |  |  |  |  |  |  |  |  |  |
| *Single* |  | 2.4 | 1 367 |  | 1.2 | 382 |  | 2.5 | 370 |
| *Co-habitant* |  | 90.2 | 50 863 |  | 95.2 | 29 216 |  | 92.1 | 13 483 |
| *Missing* |  | 7.4 | 4 162 |  | 3.6 | 1 105 |  | 5.3 | 782 |
| **Amniotic membranes status at admisson** |  |  |  |  |  |  |  |  |  |
| *Intact* |  | 68.4 | 38 600 |  | 76.7 | 23 547 |  | 77.8 | 11 392 |
| *Ruptured* |  | 29.7 | 16 723 |  | 21.5 | 6 588 |  | 20.4 | 2 980 |
| *Missing* |  | 1.9 | 1 069 |  | 1.8 | 568 |  | 1.8 | 263 |
| **Cx* dilation at admission** median 10^th^,90^th^) | 4 (3,8) |  | 56 392 | 5 (3,8) |  | 30 703 | 5 (3,8) |  | 14 635 |
| **Cx* exams during labor** median (10^th^,90^th^) | 4 (2,7) |  | 56 392 | 3 (2,5) |  | 30 703 | 3 (2,5) |  | 14 635 |
| **Oxytocin** (%) |  |  |  |  |  |  |  |  |  |
| *No* |  | 52.7 | 29 701 |  | 77.4 | 23 753 |  | 74.8 | 10 951 |
| *Yes* |  | 47.3 | 26 691 |  | 22.6 | 6 950 |  | 25.2 | 3 684 |
| **Epidural** (%) |  |  |  |  |  |  |  |  |  |
| *No* |  | 39.7 | 22 400 |  | 67.3 | 20 674 |  | 77.1 | 11 288 |
| *Yes* |  | 60.3 | 33 992 |  | 32.7 | 10 029 |  | 22.9 | 3 347 |
| **Mode of birth** (%) *Cesarean delivery* |  | 11.4 | 6 451 |  | 2.1 | 640 |  | 1.6 | 241 |
| *Non-instrumental vaginal birth* |  | 70.9 | 39 992 |  | 94.5 | 29 026 |  | 96.3 | 14 090 |
| *Instrumental delivery* |  | 17.6 | 9 949 |  | 3.4 | 1 037 |  | 2.1 | 304 |
| **Gestational length at birth** mean (SD) | 40.4 (1.21) |  | 56 392 | 40.2 (1.17) |  | 30 703 | 40.1 (1.24) |  | 14 635 |
| **Birth weight in grams** mean (SD) | 3 530 (461.2) |  | 56 360 | 3 664 (470.99) |  | 30 681 | 3 675(492.81) |  | 14 629 |
| **Head circumference in cm** mean (SD) | 34.9 (1.77) |  | 56 129 | 35 (1.77) |  | 30 611 | 35 (1.66) |  | 14 587 |
| **Fetal position** (%) |  |  |  |  |  |  |  |  |  |
| *Occiput anterior* |  | 93.9 | 52 980 |  | 95.8 | 29 428 |  | 96.2 | 14 082 |
| *Occiput posterior* |  | 6.1 | 3 412 |  | 4.2 | 1 275 |  | 3.8 | 553 |
