# Supplementary material for: First stage progression in women with spontaneous onset of labor: A large population-based cohort study
Source: PLoS One. 2020 Sep 25;15(9):e0239724. doi: 10.1371/journal.pone.0239724 (PMC7518577; doi:10.1371/journal.pone.0239724)
Supplement: S2 Table — (DOCX) [file pone.0239724.s002.docx]

**S2 Table. Duration of labor in hours from one-centimetre dilation to the next, by parity for Target population cohort**

|  |  | **Parity 0** |  | **Parity 1** |  | **Parity 2+** |  |
| --- | --- | --- | --- | --- | --- | --- | --- |
| **Cervical**  **dilation cm** | **Cervical dilation cm** | **Duration in hours (min-max)** | **n** | **Duration in hours (min-max)** | **n** | **Duration in hours (min-max)** | **n** |
| 3 | 4 | 0.94 (0.15 - 5.70) | 13 152 | 0.47 (0.06 - 4.73) | 4 400 | 0.59 (0.07 - 5.21) | 2 152 |
| 4 | 5 | 1.02 (0.17 – 6.18) | 24 025 | 0.42 (0.04 - 4.17) | 8 980 | 0.45 (0.04 - 4.76) | 4 113 |
| 5 | 6 | 0.78 (0.11 - 5.32) | 25 236 | 0.24 (0.02 - 3.10) | 10 270 | 0.20 (0.01 - 3.44) | 4 454 |
| 6 | 7 | 0.56 (0.07 - 4.42) | 23 070 | 0.12 (0.01 - 2.45) | 9 976 | 0.11 (0.0 - 2.47) | 4 135 |
| 7 | 8 | 0.38 (0.03 - 4.03) | 20 794 | 0.08 (0.00 - 2.10) | 9 359 | 0.05 (0.00 - 2.00) | 3 717 |
| 8 | 9 | 0.29 (0.02 - 3.42) | 19 954 | 0.04 (0.00 - 1.71) | 9 025 | 0.02 (0.00 - 1.35) | 3 500 |
| 9 | 10 | 0.22 (0.02 - 3.04) | 17 690 | 0.02 (0.00 - 1.26) | 6 546 | 0.01 (0.00 - 1.14) | 2 357 |

Data reported as median hours (5^th^ and 95^th^ percentiles)
