# Supplementary material for: First stage progression in women with spontaneous onset of labor: A large population-based cohort study
Source: PLoS One. 2020 Sep 25;15(9):e0239724. doi: 10.1371/journal.pone.0239724 (PMC7518577; doi:10.1371/journal.pone.0239724)
Supplement: S3 Table — (DOCX) [file pone.0239724.s003.docx]

**S3 Table. Cumulative duration of labor in hours in Para 0, 1, and 2+ based on the cervical dilation at admission for Target population cohort**

|  |  | **Parity 0** |  | **Parity 1** |  | **Parity 2+** |  |
| --- | --- | --- | --- | --- | --- | --- | --- |
| **Cervical**  **dilation cm** | **Cervical dilation cm** | **Duration in hours**  **(min -max)** | **n** | **Duration in hours**  **(min -max)** | **n** | **Duration in hours**  **(min -max)** | **n** |
| 3 | 4 | 0.92 (0.15 – 5.52) | 11 193 | 0.52 (0.06 - 4.61) | 3 954 | 0.59 (0.07 - 4.95) | 2 347 |
| 3 | 5 | 2.86 (0.85 – 9.78) | 11 143 | 1.76 (0.38 – 8.05) | 3 948 | 1.85 (0.40 - 8.51) | 2 343 |
| 3 | 6 | 4.22 (1.44 - 12.35) | 11 179 | 2.60 (0.69 - 9.85) | 3 956 | 2.61 (0.69 - 9.87) | 2 353 |
| 3 | 7 | 5.06 (1.80 - 14.21) | 11 168 | 3.01 (0.85 - 10.73) | 3 957 | 3.02 (0.84 - 10.80) | 2 355 |
| 3 | 8 | 5.68 (2.06 - 15.71) | 11 176 | 3.30 (0.96–11.36) | 3 956 | 3.22 (0.92 - 11.36) | 2 358 |
| 3 | 9 | 6.20 (2.28 - 16.90) | 11 201 | 3.50 (1.03 - 11.85) | 3 958 | 3.39 (0.98 - 11.70) | 2 361 |
| 3 | 10 | 6.65 (2.45 - 18.08) | 11 243 | 3.63 (1.08 - 12.29) | 3 964 | 3.47 (1.01–11.95) | 2 361 |
|  |  |  |  |  |  |  |  |
| 4 | 5 | 1.01(0.17- 6.02) | 15 057 | 0.41 (0.04 - 4.02) | 6 619 | 0.47 (0.05 - 4.75) | 3 440 |
| 4 | 6 | 2.51 (0.67 – 9.46) | 15 062 | 1.25 (0.23 - 6.70) | 6 621 | 1.28 (0.23- 7.19) | 3 440 |
| 4 | 7 | 3.56 (1.12 - 11.45) | 15 100 | 1.85 (0.42- 8.90) | 6 617 | 1.82 (0.40 - 8.28 | 3 447 |
| 4 | 8 | 4.26 (1.40 – 12.95) | 15 134 | 2.18 (0.53- 9.00) | 6 624 | 2.12 (0.51 - 8.88) | 3 453 |
| 4 | 9 | 4.85 (1.65 - 14.27) | 15 160 | 2.44 (0.61 - 9.70) | 6 634 | 2.32 (0.57 - 9.31) | 3 457 |
| 4 | 10 | 5.33 (1.83- 15.50) | 15 217 | 2.58 (0.65 - 10.18) | 6 640 | 2.43 (0.61 - 9.76) | 3 460 |
|  |  |  |  |  |  |  |  |
| 5 | 6 | 0.72 (0.10 – 5.19) | 9 656 | 0.22 (0.02 - 2.91) | 5 683 | 0.23 (0.01- 3.31) | 2 606 |
| 5 | 7 | 1.80 (0.40 – 8.10) | 9 651 | 0.72 (0.10 - 5.19) | 5 691 | 0.70 (0.08 - 5.67) | 2 608 |
| 5 | 8 | 2.69 (0.71 – 10.19) | 9 671 | 1.18 (0.21 - 6.62) | 5 686 | 1.13 (0.18 - 6.92) | 2 610 |
| 5 | 9 | 3.36 (0.95 – 11.86) | 9 685 | 1.48 (0.30 - 7.39) | 5 694 | 1.40 (0.25 - 7.62) | 2 611 |
| 5 | 10 | 3.90 (1.14 - 13.36) | 9 743 | 1.64 (0.34 - 7.95) | 5 705 | 1.53 (0.28 - 8.14) | 2 618 |
|  |  |  |  |  |  |  |  |
| 6 | 7 | 0.46 (0.05- 4.18) | 6 507 | 0.12 (0.01 - 2.33) | 4 718 | 0.10 (0.01 - 2.22) | 2 611 |
| 6 | 8 | 1.28 (0.22 - 7.32) | 6 494 | 0.47 (0.05 - 4.49) | 5 715 | 0.41 (0.04 - 4.29) | 2 618 |
| 6 | 9 | 2.19 (0.50- 9.56) | 6 532 | 0.86 (0.13 - 5.87) | 4 724 | 0.72 (0.09 - 5.42) | 2 014 |
| 6 | 10 | 2.80 (0.69–11.42) | 6 585 | 1.07 (0.17- 6.64) | 4 735 | 0.87 (0.12- 6.05) | 2 023 |
