# Supplementary figures and images for: First stage progression in women with spontaneous onset of labor: A large population-based cohort study
Source: PLoS One. 2020 Sep 25;15(9):e0239724. doi: 10.1371/journal.pone.0239724 (PMC7518577; doi:10.1371/journal.pone.0239724)

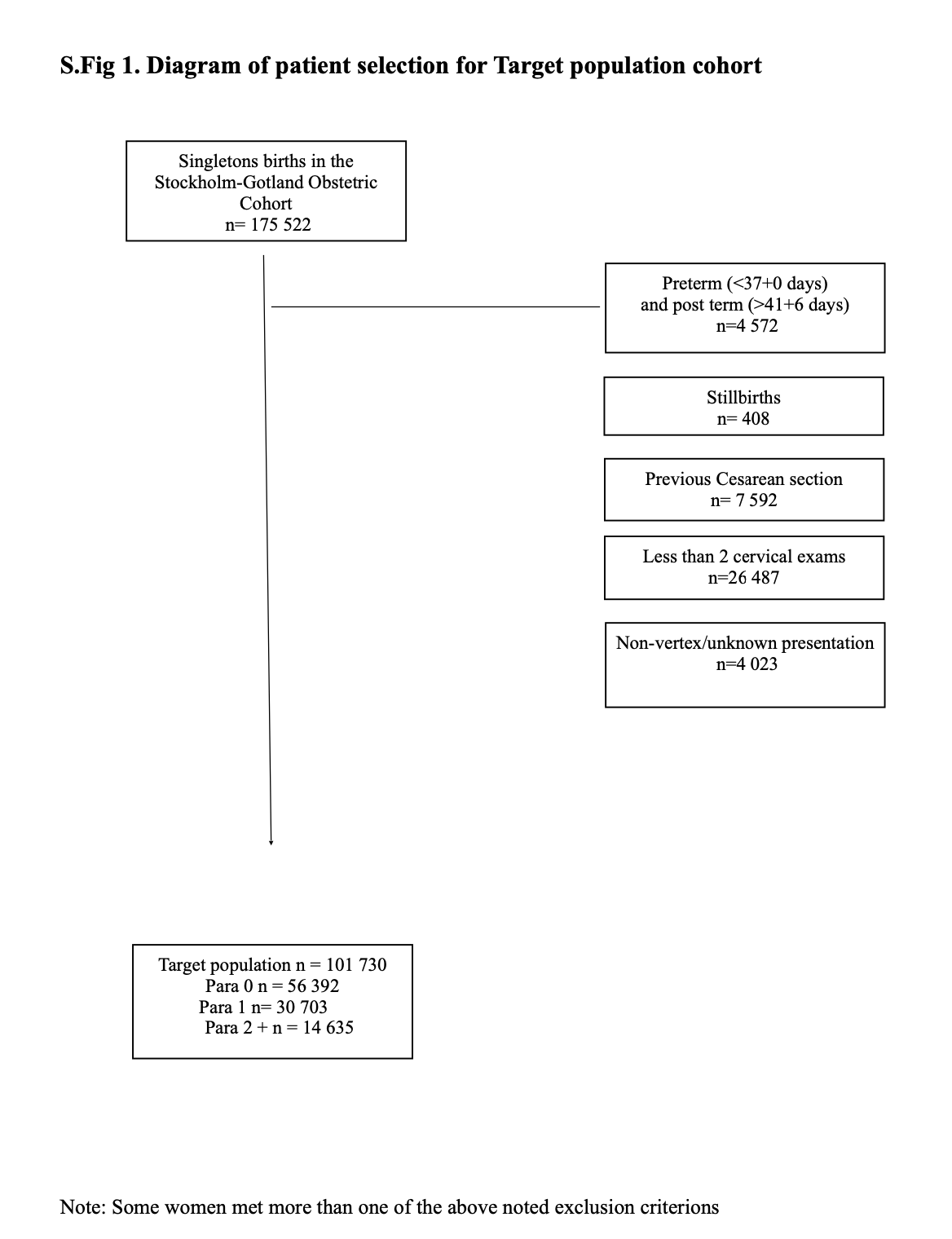

Supplement: S1 Fig — (TIF) [file pone.0239724.s004.tif]

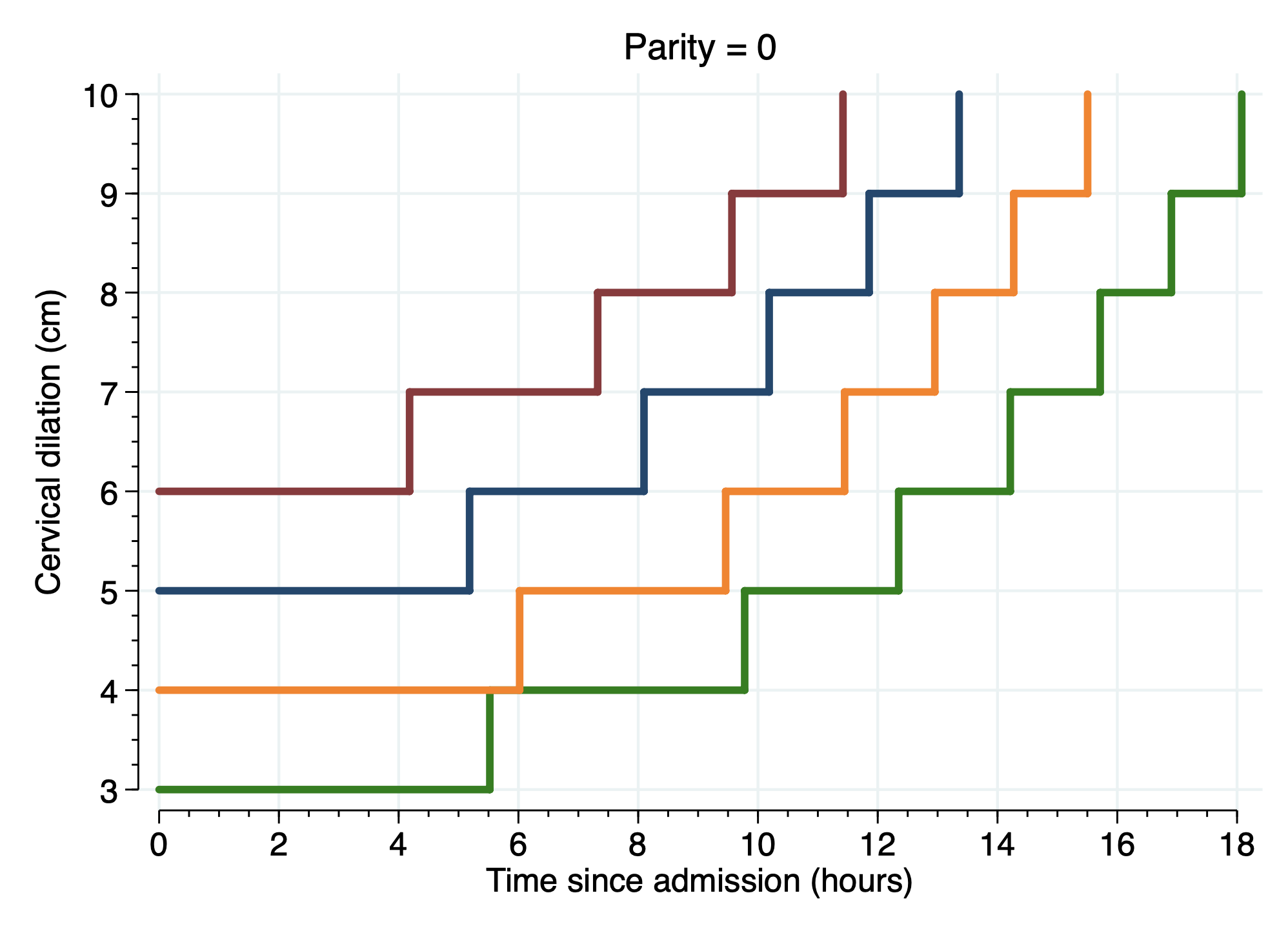

Supplement: S2 Fig — (TIF) [file pone.0239724.s005.tif]

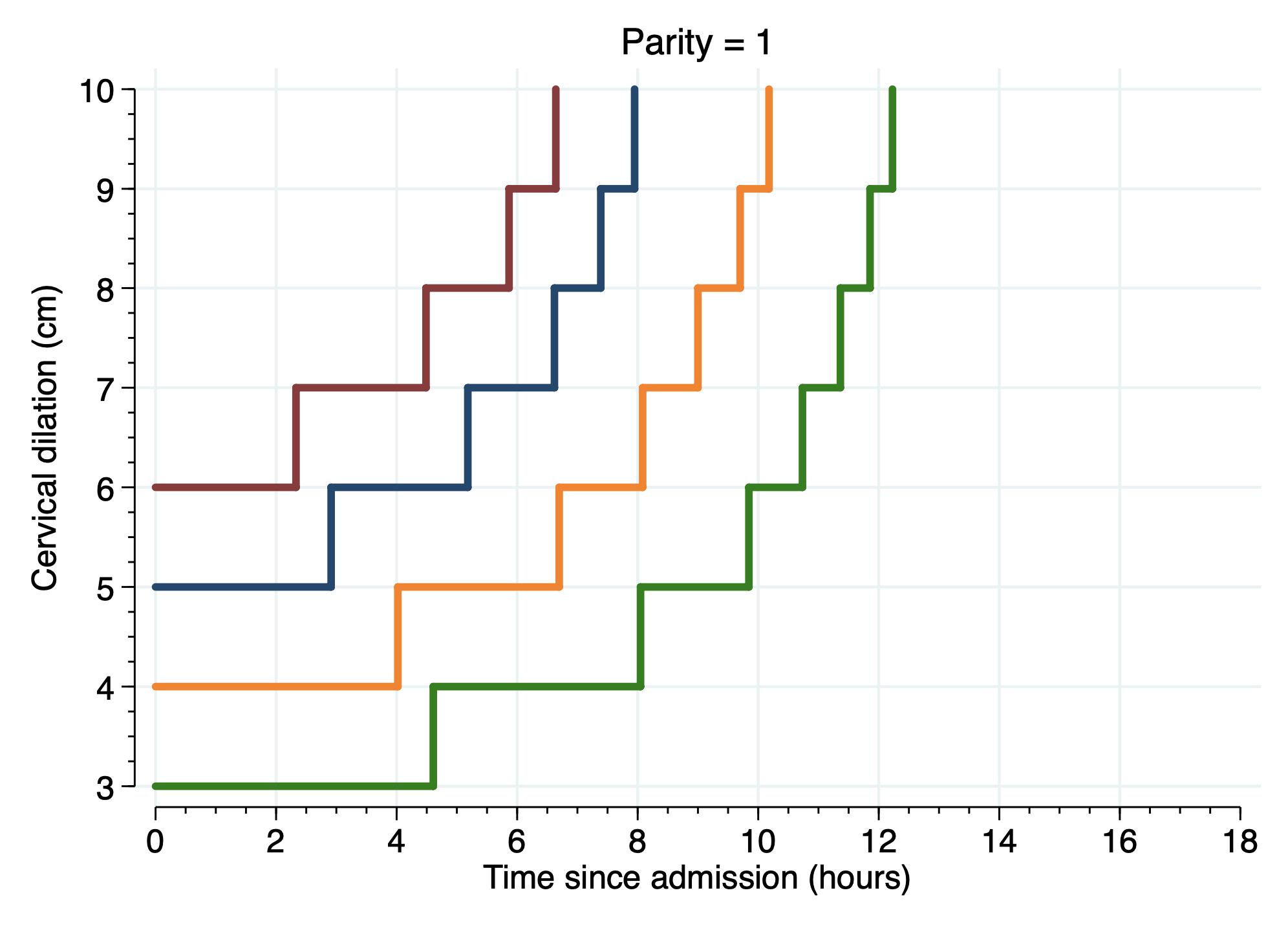

Supplement: S3 Fig — (TIF) [file pone.0239724.s006.tif]

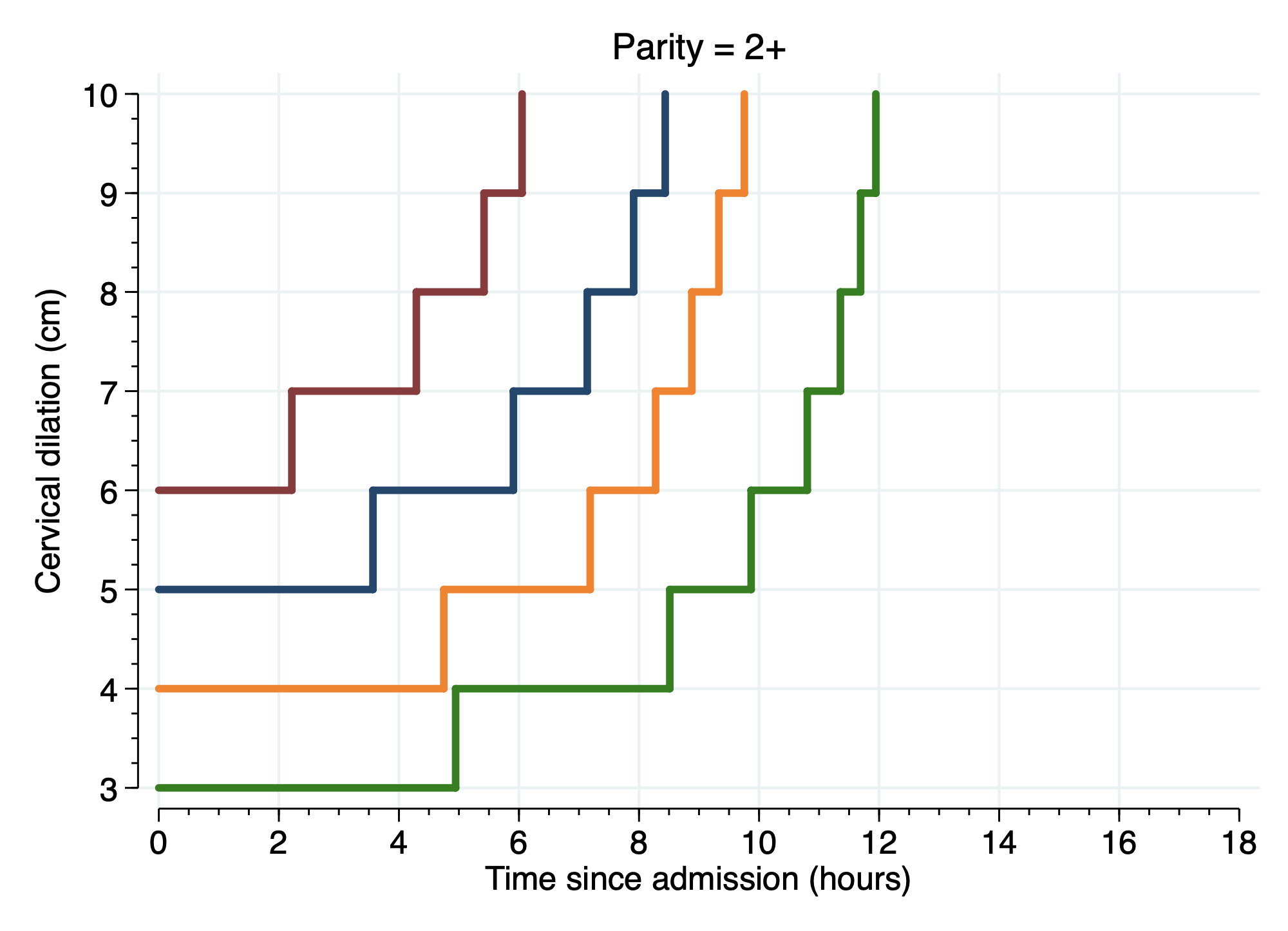

Supplement: S4 Fig — (TIF) [file pone.0239724.s007.tif]
